# Comparison of *Enterococcus* Species Diversity in Marine Water and Wastewater Using Enterolert and EPA Method 1600

**DOI:** 10.1155/2013/848049

**Published:** 2013-06-10

**Authors:** Donna M. Ferguson, John F. Griffith, Charles D. McGee, Stephen B. Weisberg, Charles Hagedorn

**Affiliations:** ^1^Southern California Coastal Water Research Project, 3535 Harbor Boulevard, Costa Mesa, CA 92626, USA; ^2^University of California, Los Angeles, Environmental Health Sciences, 650 Charles E. Young Dr. S, Los Angeles, CA 90095, USA; ^3^Orange County Sanitation District, 10844 Ellis Avenue, Fountain Valley, CA 92709, USA; ^4^Virginia Tech, Department of Crop & Soil Environmental Sciences, 330 Smyth Hall, Blacksburg, VA 24061, USA

## Abstract

EPA Method 1600 and Enterolert are used interchangeably to measure *Enterococcus* for fecal contamination of public beaches, but the methods occasionally produce different results. Here we assess whether these differences are attributable to the selectivity for certain species within the *Enterococcus* group. Both methods were used to obtain 1279 isolates from 17 environmental samples, including influent and effluent of four wastewater treatment plants, ambient marine water from seven different beaches, and freshwater urban runoff from two stream systems. The isolates were identified to species level. Detection of non-*Enterococcus* species was slightly higher using Enterolert (8.4%) than for EPA Method 1600 (5.1%). *E. faecalis* and *E. faecium*, commonly associated with human fecal waste, were predominant in wastewater; however, Enterolert had greater selectivity for *E. faecalis*, which was also shown using a laboratory-created sample. The same species selectivity was not observed for most beach water and urban runoff samples. These samples had relatively higher proportions of plant associated species, *E. casseliflavus* (18.5%) and *E. mundtii* (5.7%), compared to wastewater, suggesting environmental inputs to beaches and runoff. The potential for species selectivity among water testing methods should be considered when assessing the sanitary quality of beaches so that public health warnings are based on indicators representative of fecal sources.

## 1. Introduction

EPA Method 1600 and Enterolert (IDEXX, Westbrook, ME, USA) are two EPA-approved methods that are often used to measure *Enterococcus* for recreational bathing water quality assessments [1]. The methods have been used interchangeably for (1) regulatory monitoring to detect possible fecal contamination of water; (2) epidemiology studies to correlate swimmer's illness rates with densities of Enterococci in water, and (3) microbial source tracking studies to reduce fecal inputs to protect public health. A number of studies have found that these two methods generally produce comparable results [2–5]. However, several authors have found that the results may be markedly different [6–9]. 

There are several reasons, other than sample variability, that may explain the inconsistency between methods. EPA Method 1600 is a membrane filtration approach, where water is passed through a membrane that is subsequently placed atop *Enterococcus *Indoxyl-*β*-D-glucoside (mEI) agar and, following incubation, examined for colonies with blue halos. Enterolert is a defined substrate methodology that measures a fluorescent endpoint based on enterococci metabolizing 4-methylumbelliferone-*β*-D-glucoside in liquid media.

Differences in the combinations of growth-controlling substrates in these media could lead to selectivity of enterococcal species and detection of nonenterococcal bacteria, including *Streptococcus* spp., *Aerococcus* spp., and *Lactococcus* spp. [10]. False positive rates have been found to vary between 4% and 26% for Enterolert [11] and between 11% and 26% for mEI agar [10]. 

Another possible reason for the occasional differences between methods would be differential selectivity for species within the *Enterococcus* group, particularly since Enterolert uses a liquid broth culture media and EPA Method 1600 uses a solid agar that will affect the growth kinetics of cells. In liquid media, faster growing bacteria can outgrow their slower counterparts, even with diluted samples [12]. In contrast, bacterial cells growing on a membrane placed atop agar media are spatially separated, providing less opportunity for competition. 

Knowledge of *Entercoccus* species distribution in urban runoff and wastewater may be useful for assessing recreational waters deemed unsafe for swimming based on enterococci water quality standards. *E. faecalis* and *E. faecium* are the two most prevalent species in human feces [12]; *E. casseliflavus* and *E. mundtii* are associated with plants and soil [13]; these species are not considered typical members of the human intestinal microflora [14]. Thus, characterizing the distribution of enterococcal species representative of fecal contamination should be conducted using methods that are not subject to species selectivity. 

Here, we test the hypothesis that EPA Method 1600 and Enterolert differ in species selectivity by examining the species composition of their isolates when both methods were used to process a common set of samples. 

## 2. Methods

Differences in species selectivity were determined using 18 environmental samples and a laboratory-created sample with known species composition. The environmental samples were collected from seven marine beach sites, two freshwater urban runoff sites, influent from four wastewater treatment plants (WWTPs), and secondary effluent from four WWTPs (Table 1). The beach water was collected using 100 mL plastic bottles at ankle depth upon an incoming wave. Urban runoff was collected as 1 L samples just below the water surface. Wastewater was collected as 1 L samples from influent or effluent pipes. 

The laboratory-created sample consisted of clean seawater inoculated with approximately 1,000 colony forming units per 100 mL each of *E. faecium *and *E. faecalis*. The seawater was collected 18 kilometers offshore, at 10-meter depth with no measurable enterococci present. The enterococci cultures were prepared using strains from environmental samples that were enumerated using EPA Method 1600 and Enterolert and identified to species using the Vitek microbial identification system (bioMérieux, St. Louis, MO, USA).

All samples were analyzed within six hours of collection following EPA standards [15] and the Enterolert manufacturer's instructions. For EPA Method 1600, 10–50 mL volumes of sample were filtered onto mEI, and presumptive enterococci isolates were obtained by selecting up to five colonies (per sample) with blue halos from mEI agar (Northeast Laboratory, Waterville, ME, USA) and subculturing them onto tryptic soy agar (TSA) with 5% sheep blood (Northeast Laboratory, Waterville, ME, USA). After 24 h incubation at 35°C, the blood agar plates (BAPs) were examined to ensure they were pure cultures. Isolates from BAPs were subcultured onto TSA slants (Northeast Laboratory, Waterville, ME, USA) and incubated as before. The TSA slants were stored at 4°C until speciation was performed. 

Ten mL of sample were used for the Enterolert Quanti-Tray method and enterococci isolates following the method of Kinzelman et al. [6]. The back of the Quanti-Tray was disinfected with 70% alcohol, and media from up to five fluorescing (positive) wells was withdrawn using sterile syringes. The media was then inoculated into brain heart infusion (BHI) broth (BD, Franklin Lakes, New Jersey, USA) containing 6.5% NaCl at a 1 : 20 dilution. Inoculated broth was then incubated at 41°C for 48 h. Cultures that had growth were subcultured onto BAPs, and then colonies from the BAPs were subcultured onto TSA slants, incubated, and stored at 4°C. 

### 2.1. Isolate Identification

Approximately 80 isolates each from 17 environmental samples that were obtained using EPA Method 1600 and Enterolert were identified to species using the Vitek microbial identification system (bioMérieux, St. Louis, MO, USA) (Table 2). Isolates identified as *Enterococcus* species with discrimination of <80% confidence were categorized as “indeterminant”. Isolates identified as species other than *Enterococcus* were categorized as “non-*Enterococcus*”. *E. casseliflavus*/*E. gallinarum* isolates that could not be discriminated using Vitek were tested for motility and pigment production following Ferguson et al. [10] to differentiate these species. Since *E. mundtii* is not identified by Vitek [16], a total of 107 isolates from beach water, wastewater and freshwater were screened for this species using published biochemical tests, including motility; pigment production; and fermentation of arabinose, sucrose, and mannitol [17]. 

## 3. Results and Discussion

### 3.1. *Enterococcus* Species Assemblages

**Figure 1:**
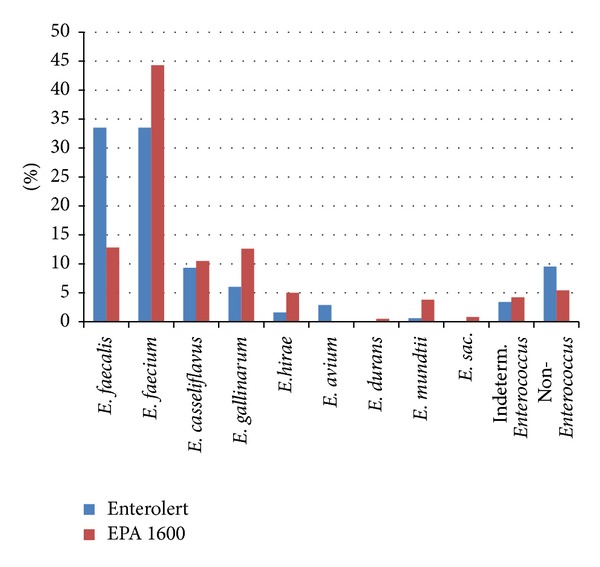
*Enterococcus *species found overall using Enterolert versus EPA Method 1600.

1279 presumptive enterococci isolates from environmental samples (beach water, urban runoff, and wastewater treatment plant influent and effluent) (Table 2) using EPA Method 1600 and Enterolert were examined and found to include nine species of *Enterococcus*. *E. faecium* and *E. faecalis* were the most frequent species identified among isolates obtained using Enterolert, with each species comprising about one third of isolates from all samples (Figure 1). These were also the most frequent species obtained using EPA Method 1600, though *E. faecium* was more common (44%) than *E. faecalis *(13%). *E. gallinarum *and* E. casseliflavus *were the next most frequently identified species by both methods. Non-*Enterococcus *species comprised 8% of the isolates derived from Enterolert and 5% from EPA Method 1600. The most common nonenterococcal bacteria isolated were *Proteus mirabilis *from Enterolert wells and* Aerococcus viridans* from EPA Method 1600. Other species identified by both methods included *Streptococcus bovis*, *S. uberis*, *S. mutans*, and *S. pneumonia*. Five percent of the isolates were identified with a low level of certainty and classified as “indeterminant.”

**Figure 2:**
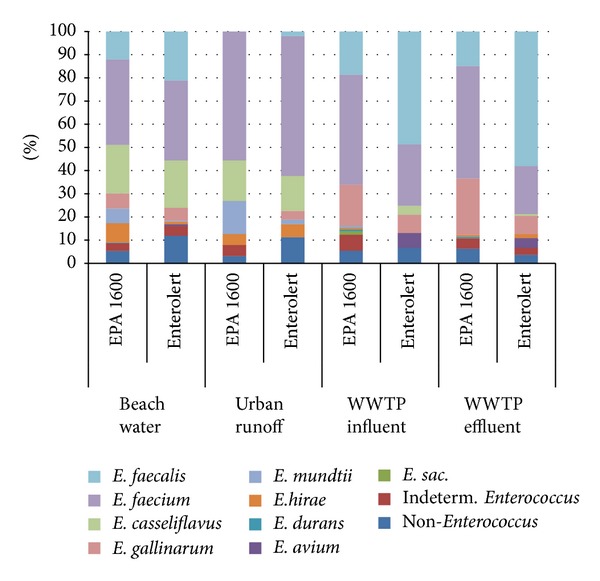
Distribution of predominant *Enterococcus* species found among beach water, urban runoff, and wastewater treatment plant (WWTP) influent (untreated) and effluent (treated) samples.

Overall relative abundance of species among all the environmental samples was not significantly different between methods (*χ* ^2^, (1, *N* = 1104) = 47.4, *P* < 0.001). However, the relative proportions of *E. faecium* and *E. faecalis* were significantly different between methods for the wastewater samples, with *E. faecalis* the most frequently observed species for Enterolert and *E. faecium* dominant for EPA Method 1600 (Figure 2). The proportions of *E. faecalis* and *E. faecium* were generally similar among two urban runoff samples and eight beach water samples (Fisher's exact test, *P* = 0.31).

**Figure 3:**
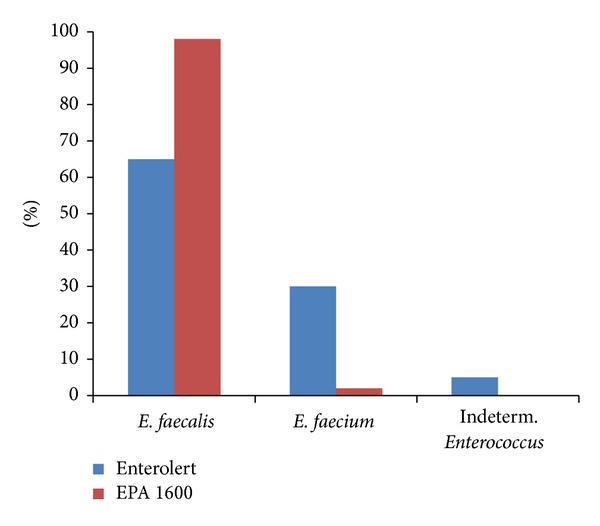
Percent *E. faecalis* and *E. faecium* isolates from culture sample containing 1 : 1 ratio of both species obtained using Enterolert and EPA Method 1600.

The same selectivity observed in the wastewater samples using Enterolert was also observed in the laboratory sample spiked with similar concentrations of *E. faecalis* and *E. faecium* (Figure 3). EPA Method 1600 identified 65% of the isolates as *E. faecalis*, 30% as *E. faecium*, and 5% as “indeterminant.” In contrast, 98% of the Enterolert isolates were identified as *E. faecalis*.

Enterococcal species identification using Vitek was supplemented with pigment production and motility testing to identify *E. mundtii* and *E. casseliflavus* that may be misidentified as *E. gallinarum* by Vitek alone [16]. Of the 107 isolates screened for *E. mundtii*, twelve isolates were misidentified as *E. gallinarum* by Vitek; six of these were confirmed as *E. mundtii*, four as *non-Enterococcus* species, and two as *E. casseliflavus* based on additional biochemical tests, pigmentation, and motility. Vitek identifications of *E. faecalis* and *E. faecium* were much more accurate; all but one isolate each of *E. faecalis* and *E. faecium* were identified similarly by Vitek and conventional biochemical tests. 

### 3.2. *Enterococcus* Species Selectivity

While the two methods generally yielded the same species assemblage, Enterolert identified a higher proportion of *E. faecalis* than EPA Method 1600 did across all sample types. There are a number of reasons why this might occur, one of which is the difference between liquid and solid growth media discussed earlier. Another difference is media formulation. The mEI media used in EPA Method 1600 contain additives, such as triphenyltetrazolium chloride to differentiate *Enterococcus* from other Gram-positive cocci, sodium azide and nalidixic acid to inhibit the growth of Gram-negative bacteria, and cycloheximide to suppress the growth of fungi; Enterolert media are proprietary, and it is unknown whether similar additives to increase specificity are present. The media also differ in the reporter molecules they use to detect enterococci. While mEI media rely on the ability of *Enterococcus* to metabolize indoxyl-*β*-D-glucoside to produce a blue compound, Enterolert media rely on 4-methylumbelliferone-*β*-D-glucoside to produce a fluorescent metabolite. 

 Another potential mechanism for the observed differences is oxygen availability. Enterolert media are heat-sealed within a Quanti-Tray, creating a more anaerobic incubation condition compared to mEI agar in a petri dish. Enterococci are facultative anaerobes [17], but it is unknown whether growth rates vary among species and strains under differing oxygen levels. Differences in growth rates among *Enterococcus* species could also account for differing results between methods. Delayed growth rates and positive fluorescence reactions (after 24 hours) of certain enterococcal strains in Quanti-Trays raised concerns that Enterolert may underestimate enterococci densities [9, 18]. Species selection bias could have been introduced during the BHI broth (with 6.5% NaCl) subculture step that we used to facilitate isolation of enterococci from Enterolert. This enrichment step was used to reduce non-*Enterococcus* species in samples and also because previous attempts to subculture directly from Enterolert onto nonselective media resulted in nondetection of enterococci. To assess potential selection bias, we compared the growth rates of *E. faecalis *(ATCC 29212), *E. faecium* (ATCC 35667), and *E. casseliflavus* (ATCC 700527) in BHI broth with 6.5% NaCl versus Enterolert media, determining the doubling time of each species using optical density (600 nanometers; UV160U Spectrophotometer, Shimadzu Scientific Instruments, Columbia, MD, USA) and plate counts on mEI. *E. faecium* grew faster than *E. faecalis* in Enterolert and in BHI broth with 6.5% NaCl. However, Enterolert was not selective for *E. faecium*, and *E. faecium* was not predominant in the actual samples suggesting that other factors likely accounted for the Enterolert selectivity for *E. faecalis* observed in the wastewater samples. Enterolert also showed the same selectivity for *E. faecalis* among isolates subcultured directly from a laboratory-created sample without using BHI step.

### 3.3. Speciation Method Limitations

We used the Vitek system, which is routinely used by environmental laboratories, to verify presumptive enterococci on mEI, for most of our species identification (APHA 2000). One limitation of Vitek is that the database does not include *E. mundtii*, which is a species that is present in environmental waters [10]. In a previous study, Moore et al. [16] found that 14% of the isolates speciated by Vitek without supplementary testing were misidentified. In our study, 17% of the isolates identified by Vitek had discrepant identifications compared to conventional biochemical testing. The majority of these were isolates that had been misidentified by Vitek as *E. gallinarum* that were later identified as *E. mundtii*, *E. casseliflavus*, or non-*Enterococcus* based on supplementary testing including pigment, motility, and additional biochemical tests. Thus, the overall percentages of *E. gallinarum* identified in this study using Vitek alone may be somewhat inflated.

Molecular methods that could have been used to identify *Enterococcus *isolates include 16S rRNA gene sequencing [18] and genus and species specific multiplex-PCR [19]. These methods are rapid, cost-effective and allow high throughput. In some cases, conventional biochemical testing may be useful when used in conjunction with molecular methods, such as 16S rRNA sequencing [20, 21]. For example, pigment production and motility can be used to discriminate *E. casseliflavus* and *E. gallinarum *with identical 16S rRNA gene sequences [16]. Since *E. casseliflavus* is associated with plants, discriminating these two species could be important for assessing natural sources. Multiplex-PCR is another molecular-based speciation method that was shown as having 90% concordance with Vitek and conventional biochemical methods for identifying Enterococcal species from environmental and fecal isolates [19]. However, the sensitivity of multiplex PCR may be a concern; Layton et al. [22] used a modified version of Jackson's multiplex PCR method and identified eight *Enterococcus* species among isolates from animal fecal samples but found that samples with less than 30 colony forming units (CFU) required a culture enrichment step; following enrichment, ~70% of potential enterococcal species were detected using multiplex PCR. 

### 3.4. *Enterococcus* Species Distribution in Wastewater, Beach Water, and Urban Runoff

While there were some differences in species selectivity between Enterolert and EPA Method 1600, these differences were much smaller than the species composition differences that both methods found among the different sample types. For instance, both methods found much higher percentages of *E. casseliflavus *in beach samples (~20%) than in wastewater samples (<4%). Both methods also found similar enterococcal species distribution among sewage influent from the four wastewater treatment plants, despite differences in size, treatment processes, and the nature of service areas. The dominance of *E. faecium* and *E. faecalis* in the wastewater systems is consistent with previous studies that have examined species distributions of *Enterococcus* in wastewater [23–25]. Higher percentages of *E. faecalis* and *E. faecium* in wastewater streams are also consistent with clinical studies that have established that these two species comprise a significant fraction of the enterococci found in human and animal feces [14, 22, 26]. Similarly, the prevalence of *E. casseliflavus* in beaches and runoff samples with minimal human fecal sources and its lower occurrence in wastewater samples is also consistent with clinical human fecal samples [27, 28] and its known association with natural sources, such as plants [29, 30]. 

## 4. Conclusions 

EPA Method 1600 and Enterolert detected similar proportions of *Enterococcus *species in marine and spiked samples; however, Enterolert was more selective for *E. faecalis* in wastewater samples. Also, Enterolert yielded higher percentages of non*-Enterococcus* organisms in beach water and runoff samples, which could account for occasional differences in water quality assessments using both methods. Further insights on the diversity of *Enterococcus *species in environmental waters may help to improve studies on health risk assessments. For species identification of *Enterococcus* using culture methods, EPA Method 1600 is recommended to obtain a more accurate characterization.

## Figures and Tables

**Table 1:** Sources of samples.

Beach water	
Imperial Beach, San Diego	
San Mateo Beach, San Clemente	
Doheny State Beach, Dana Point	
Cabrillo Beach, Los Angeles	
Surfrider Beach, Malibu	
Paradise Cove, Malibu	
Big Sycamore, Malibu	
Urban runoff	
Dominguez Channel, Los Angeles	
Tijuana River, San Diego	
Wastewater treatment plant (WWTP)	
Joint Water Pollution Control Plant of the Los Angeles County Sanitation District	
Orange County Sanitation District, Huntington Beach	
South Orange County Wastewater Authority, Dana Point	
Encina Wastewater Authority, Carlsbad	

**Table 2:** Sources of samples and numbers of isolates analyzed for speciation using EPA Method 1600 and Enterolert.

Source (no. of samples)	EPA Method 1600	Enterolert	No. of isolates
Beaches (7)	275	303	578
Urban runoff (2)	91	99	290
Wastewater influent (4)	126	130	256
Wastewater effluent (4)	129	126	255
Culture (1)	20	46	66

Total	621	658	1279
